# Vitamin D Deficiency Induces High Blood Pressure and Accelerates Atherosclerosis in Mice

**DOI:** 10.1371/journal.pone.0054625

**Published:** 2013-01-22

**Authors:** Sherry Weng, Jennifer E. Sprague, Jisu Oh, Amy E. Riek, Kathleen Chin, Miguel Garcia, Carlos Bernal-Mizrachi

**Affiliations:** 1 Department of Anesthesiology, Washington University, St. Louis, Missouri, United States of America; 2 Division of Pediatric Endocrinology and Diabetes, Washington University, St. Louis, Missouri, United States of America; 3 Division of Endocrinology, Metabolism, and Lipid Research, Washington University, St. Louis, Missouri, United States of America; 4 Department of Cell Biology and Physiology, Washington University, St. Louis, Missouri, United States of America; State University of Rio de Janeiro, Biomedical Center, Institute of Biology, Brazil

## Abstract

Multiple epidemiological studies link vitamin D deficiency to increased cardiovascular disease (CVD), but causality and possible mechanisms underlying these associations are not established. To clarify the role of vitamin D-deficiency in CVD in vivo, we generated mouse models of diet-induced vitamin D deficiency in two backgrounds (LDL receptor- and ApoE-null mice) that resemble humans with diet-induced hypertension and atherosclerosis. Mice were fed vitamin D-deficient or -sufficient chow for 6 weeks and then switched to high fat (HF) vitamin D-deficient or –sufficient diet for 8–10 weeks. Mice with diet-induced vitamin D deficiency showed increased systolic and diastolic blood pressure, high plasma renin, and decreased urinary sodium excretion. Hypertension was reversed and renin was suppressed by returning chow-fed vitamin D-deficient mice to vitamin D-sufficient chow diet for 6 weeks. On a HF diet, vitamin D-deficient mice had ∼2-fold greater atherosclerosis in the aortic arch and ∼2–8-fold greater atherosclerosis in the thoracic and abdominal aorta compared to vitamin D-sufficient mice. In the aortic root, HF-fed vitamin D-deficient mice had increased macrophage infiltration with increased fat accumulation and endoplasmic reticulum (ER) stress activation, but a lower prevalence of the M1 macrophage phenotype within atherosclerotic plaques. Similarly, peritoneal macrophages from vitamin D-deficient mice displayed an M2-predominant phenotype with increased foam cell formation and ER stress. Treatment of vitamin D-deficient mice with the ER stress reliever PBA during HF feeding suppressed atherosclerosis, decreased peritoneal macrophage foam cell formation, and downregulated ER stress proteins without changing blood pressure. Thus, we suggest that vitamin D deficiency activates both the renin angiotensin system and macrophage ER stress to contribute to the development of hypertension and accelerated atherosclerosis, highlighting vitamin D replacement as a potential therapy to reduce blood pressure and atherosclerosis.

## Introduction

Atherosclerosis-related events remain a leading cause of mortality in the United States with coronary artery disease (CAD) accounting for approximately 1 of every 6 deaths [1]. Dyslipidemia is a major risk factor for CAD. However, aggressive low density lipoprotein (LDL) cholesterol lowering only reduces all-cause mortality and major coronary events by ∼ 30% [2]. Monocyte-derived macrophages play a pivotal role in vascular lipid deposition and the subsequent progression of atherosclerosis [3]. Macrophages recruited to the subendothelial space respond to environmental signals that dictate their differentiation into different phenotypes with diverse cholesterol metabolism and functional programs. Both classically-activated (M1) and alternatively-activated (M2) macrophages are found within human atherosclerotic plaques [4]. In the atherosclerotic plaque, interferon (IFN)γ induces M1 macrophages which are characterized by the generation of proinflammatory cytokines that accelerate additional immune cell recruitment, but this cell subtype also expresses membrane receptors that facilitate plaque macrophage egression [5]–[10]. In contrast, M2 macrophages induced by interleukin (IL)-4, IL-10, or immunocomplex are known to attenuate excessive inflammation, facilitating collagen production and fibrosis, but have a higher capacity to accumulate lipids [6], [11]–[13]. Therefore, understanding the mechanisms that control macrophage phenotype plasticity to suppress lipid accumulation is a key factor in atherosclerosis prevention and regression.

Endoplasmic reticulum (ER) stress activation is an increasingly recognized factor in the development of atherosclerosis [14]. Cells attempt to relieve ER disturbance by activating ER stress signaling pathways (unfolded protein response) to attenuate global protein translation and degrade unfolded proteins. Monocyte differentiation into macrophages triggers structural and functional reorganization of the ER to perform the new cellular functions, thus leading to ER stress and upregulation of the unfolded protein response [15]–[18]. In murine models of diet-induced atherosclerosis, ER stress is detected in intimal macrophages at early stages of vascular inflammation [19]. In humans and mice, ER stress is also present in advanced atherosclerotic plaques leading to accelerated macrophage apoptosis and increased plaque vulnerability which may lead to acute coronary syndrome [14], [20]–[22]. Knock-out of the ER stress protein CEBP homologous protein (CHOP) or treatment with chemical chaperones to decrease ER stress prevents the development of atherosclerosis [23], [24]. Moreover, in patients with diabetes, we have shown that the suppression of ER stress shifts M2-predominant macrophages to M1-predominant cells and decreases foam cell formation [25]. Therefore, discovering systemic and local environmental conditions that modulate ER stress in the vessel wall will be critical to the development of new therapeutic targets to regulate macrophage phenotype, cholesterol deposition, and plaque progression.

Vitamin D deficiency is a largely unacknowledged epidemic associated with cardiovascular disease (CVD) risk and its associated mortality [26]–[28]. Approximately 1 billion people worldwide have low levels of 25-hydroxyvitamin D [25(OH)D], the principal circulating storage form of vitamin D, and 25(OH)D deficiency (<37 nmol/L) is independently associated with cardiovascular events in patients with hypertension [29], [30]. Macrophages, endothelial cells, and smooth muscle cells, among others, are also able to transform 25(OH)D_3_ to its active hormonal form, 1,25-dihydroxy vitamin D [1,25(OH)_2_D]. This increased local production of active vitamin D serves as an autocrine/paracrine factor, which is fundamental for cell-specific functions [31]. A growing body of evidence from animal and human studies illustrates that vitamin D decreases systemic inflammatory mediators of vascular disease and imbues immune cells with anti-inflammatory properties [32]–[34]. In diabetics, we have shown that 1,25(OH)_2_D suppresses cholesterol uptake, prevents foam cell formation, and reverses cholesterol deposition in macrophages by downregulation of ER stress [35]. Furthermore, suppression of ER stress by 1,25(OH)_2_D shifts M2-predominant macrophages to M1-predominant cells, suggesting that regulation of ER stress by vitamin D could be a potential therapy for atherosclerosis. In mice, the absence of vitamin D receptor signaling increases blood pressure by increasing renal renin release and accelerates atherogenesis possibly by local activation of the renin angiotensin system (RAS) in macrophages [36]. However, these models are limited because they do not mimic the in vivo vitamin D deficiency commonly seen in humans. The effects of diet-induced vitamin D deficiency on atherosclerosis remain uncertain.

In this study, we utilized dietary intervention in animal models of atherosclerosis to mimic human vitamin D deficiency and sufficiency and evaluated whether vitamin D deficiency induces hypertension and accelerates atherosclerosis. Additionally, we investigated whether vitamin D status regulates macrophage phenotype and ER stress activation as possible mechanisms underlying the vascular effects of vitamin D.

## Materials and Methods

### Animals

This study was carried out in strict accordance with the recommendations in the Guide for the Care and Use of Laboratory Animals of the National Institutes of Health. The protocol was approved by the Washington University Animal Studies Committee (Permit Number: 20120167). LDL receptor knock-out (LDLR^−/−^) and apolipoprotein E knock-out (ApoE^−/−^) mice were weaned to either standard chow (chow_D+_, HarlanTD 7022) or vitamin D-deficient chow with 2% calcium (chow_D−_, Harlan TD87095), both providing 5.2% calories as fat. After 6 weeks (baseline), mice were subsequently fed a high fat diet (HFD) which was vitamin D-sufficient (HFD_D+_, Harlan TD88137) or vitamin D-deficient with 2% calcium (HFD_D−_, Harlan TD07019), both containing 0.15% cholesterol with 42% calories as fat. For atherosclerosis experiments, mice were fed HFD for 8 weeks for ApoE^−/−^ and 10 weeks for LDLR^−/−^. Additional LDLR^−/−^ mice were maintained on chow_D+_ or chow_D−_ from weaning for 1 year prior to sacrifice for atherosclerosis assessment. Isoflurane anesthesia was used for retro-orbital venous plexus puncture and sacrifice by cervical dislocation. All efforts were made to minimize suffering. All experiments included both male and female animals.

### Metabolic Assessment

Mice underwent metabolic characterization at baseline (6 weeks after weaning) and after HFD. Serum 25(OH)D and 1,25(OH)_2_D levels were determined by radioimmunoassay (DiaSorin, Inc.; Stillwater, MN), and serum calcium levels were determined by atomic absorption spectrophotometry (Perkin-Elmer, model 1100B, Norwalk, CT). Serum analysis for glucose, cholesterol, triglycerides, and free fatty acids was carried out as described previously [37]. Body composition was determined by magnetic resonance imagine (MRI) of conscious mice according to the manufacturer’s protocol (EchoMRI 3-1, Echo Medical Systems).

### Blood Pressure

Systolic blood pressure (SBP) and diastolic blood pressure (DBP) were measured in conscious mice using a tail-cuff system (Kent Scientific) at baseline and after HFD as we previously described [38], [39]. Animals (LDLR^−/−^ and ApoE^−/−^) were acclimated to handling and placement in the apparatus daily for 3 days before the measurement of blood pressure. Blood pressures were also measured on LDLR^−/−^ mice maintained on chow_D−_ for 6 weeks and then returned to chow_D+_ for 6 weeks (vitamin D replacement) to evaluate blood pressure reversibility. Noninvasive results were confirmed in a subset of ApoE^−/−^ mice after HFD using a PowerLab/8SP invasive monitoring instrument (AD Instruments) as previously described [38]. Twenty-four hour urine collections for urinary sodium were obtained using metabolic cages as previously described [40]. Renin activity was measured in plasma samples pooled from groups of five animals by radioimmunoassay of in vitro-generated angiotensin I using a kit from DiaSorin.

### Mouse Atherosclerotic Lesions

For atherosclerosis assays, aortae were prepared using the *en face* technique [38], [41]. Results were reported as percentage involvement of the intimal surface for three regions of the aorta as analyzed using ImageJ. To detect the colocalization of differentiated macrophages with cholesterol deposition in the atherosclerotic plaque in vivo, we stained 10-µm serial cryosections of the aortic root from ApoE^−/−^ mice after HFD with antibodies specific for chemokine CC motif receptor 7 (CCR7, M1 marker) or manose receptor (MR, M2 marker) (1∶100 for both, Santa Cruz Biotechnology) and adipocyte differentiation-related protein (ADRP, 1∶100, American Research Product, Belmont, MA) following the manufacturer’s recommendations as we previously described [25]. Plaque M1 or M2 macrophages were measured by the percentage of total plaque area (determined based on adjacent H&E-stained sections) with staining for the membrane receptor for each phenotype. Lipid colocalization with M1 or M2 macrophages was measured by the percentage of total ADRP staining area colocalizing with staining for the membrane receptor for each phenotype.

### Isolation of Murine Peritoneal Macrophages

Unstimulated peritoneal macrophages were collected from HFD-fed mice immediately following injection of 10 mL of phosphate buffered saline into the peritoneum for evaluation of cell surface markers and ER stress protein expression. In order to collect adequate cells for cholesterol metabolism analysis, peritoneal macrophages were isolated from HFD-fed mice 3 days after intraperitoneal injection of 4% thioglycollate solution, as previously described [25], [42]. Isolated macrophages (0.5×10^6^ cells per well in 12-well plates) were cultured in vitamin D-deficient or 1,25(OH)_2_D_3_-supplemented (10^−8^ M) media for 3 hours for stabilization (except for cholesterol uptake and efflux, described below) prior to assessment, as we have previously described [25].

### Macrophage Cholesterol Metabolism

Foam cell formation (Oil-Red-O stain), cholesterol uptake, and efflux were assessed as we previously described [25], [35]. Foam cell formation was assessed by fixing macrophage slides with 5% paraformaldehyde for 15 minutes and staining with Oil-red-O. To assess cholesterol uptake, macrophages were incubated for 6 hours at 37°C with with 10 µg/mL oxidized LDL (oxLDL) labeled with 1,1′-dioctadecyl-3,3,3′,3′-tetramethyl indocarbocyanine percholate (DiI; Invitrogen). For cholesterol efflux, peritoneal macrophages were incubated for 24 hours with oxLDL (100 µg/mL) preincubated with 5 mCi of ^3^H cholesterol (American Radiolabeled Chemical, Inc.). Six hours following replacement of media with serum-free media containing apolipoprotein AI (25 µg/mL) or HDL (50 µg/mL), supernatant and cells were assessed for radioactivity. Efflux of ^3^H cholesterol from the cells into the medium was calculated as percent of total ^3^H cholesterol incorporated in the cells after incubation. Lipids (total and free cholesterol, triglycerides) from peritoneal macrophages were extracted with chloroform/methanol (2∶1 v/v), dried under nitrogen, and reconstituted for enzymatic assays using commercial reagents (Thermo Electron Corp, Waltham, MA) [25]. Results were normalized to total cell protein concentrations.

### Flow Cytometry

Macrophage cell surface marker analysis was performed using a FACStar Plus with PE-conjugated anti-CCR7 and anti-CD86 (1∶100 for both, E-Bioscience) for M1 macrophage membrane protein expression and FITC-conjugated anti-CD163 (1∶20, Bioss USA) and anti-MR (1∶100, R&D Systems) for M2 macrophage membrane protein expression [25]. Macrophage phenotype ratio was calculated as (CCR7+ CD86 expression)/(CD163+ MR expression), all expressed as percentage of cells positive, to determine the relative abundance of M1 and M2 cell subtypes [43].

### Assessment of ER Stress in Vivo

Protein lysates were analyzed by Western blot for ER stress protein expression [anti-pPERK (1∶1000, Cell Signaling), anti-pIRE1α (1∶500, AbCAM), anti-CHOP (1∶500, Santa Cruz)]. Western blot analyses were normalized to β-actin (1∶1000, Cell Signaling) expression. To detect macrophage ER stress in the atherosclerotic plaque in vivo, we stained 10-µm serial cryosections of the aortic root from ApoE^−/−^ mice after HFD with antibodies specific for macrophage MOMA or ER stress chaperone protein CHOP (1∶200 for both, Santa Cruz) as we previously described [25]. To suppress ER stress, ApoE^−/−^ mice at 12 weeks post-weaning received treatment with 4-phenyl butyric acid (PBA, Sigma), 1 g/kg/day, or control saline by gavage twice daily as previously described for 8 weeks while on HFD_D−_ [44]. During the last week of treatment, blood pressure and metabolic profiles were assessed. Mice were then sacrificed for peritoneal macrophage and atherosclerosis analysis as described above.

### Statistical Analysis

Experiments were carried out with duplicate or triplicate samples. Parametric data are expressed as mean ± standard error of the mean and analyzed by *t-*tests. Non-parametric data (atherosclerosis) are presented as median and analyzed using the Mann-Whitney test. Differences were considered statistically significant if *p*≤0.05. Statistical analysis was carried out using GraphPad Prism.

## Results

### Vitamin D Deficiency does not Alter Weight or Metabolic Profile

To induce a vitamin D-deficient state, 3-week-old mice (ApoE^−/−^ and LDLR^−/−^) were fed standard chow diet (chow_D+_) or vitamin D-deficient diet (chow_D−_) for six weeks (baseline) before starting either standard vitamin D-sufficient high fat diet (HFD_D+_) or vitamin D-deficient high fat diet (HFD_D−_). We studied two different murine models of atherosclerosis to ensure that the effects of vitamin D were not background-specific because LDR^−/−^ and ApoE^−/−^ mice have differences in their susceptibility to develop dyslipidemia, diet-induced obesity, and type 2 diabetes phenotypes [45], [46]. Mice were 25(OH)D-deficient after 4 weeks on deficient chow (chow_D−_ 9.2±0.7 nmol/L vs. chow_D+_89.4±14.2 nmol/L, p<0.005), and remained deficient on HFD (HFD_D−_ 19.9±3.5 nmol/L vs. HFD_D+_59.8±11.7 nmol/L, p<0.005). Serum 1,25(OH)_2_D levels were not different (HFD_D−_ 198±22 pmol/L vs. HFD_D+_214±28 pmol/L, p<0.7). Serum calcium remained unchanged after vitamin D-deficient chow or HFD when compared to standard diets (chow_D−_ 9.1±0.4, chow_D+_9.2±0.1, HFD_D−_ 9.7±0.3, HFD_D+_9.4±0.3, n = 4 per group). Vitamin D deficiency did not affect total body weight or percent body fat as assessed by MRI at baseline or after HFD (Figures S1, S2). To determine whether vitamin D deficiency induces metabolic changes, metabolic profiles were compared for vitamin D-deficient and -sufficient mice after HFD or LDLR^−/−^ mice after 1 year on chow diet (Figures S1, S2) There were no differences in blood glucose, total cholesterol, triglycerides, or free fatty acids.

### Vitamin D Deficiency Reversibly Increases Blood Pressure by Modulation of the Renin Angiotensin System

**Figure 1:**
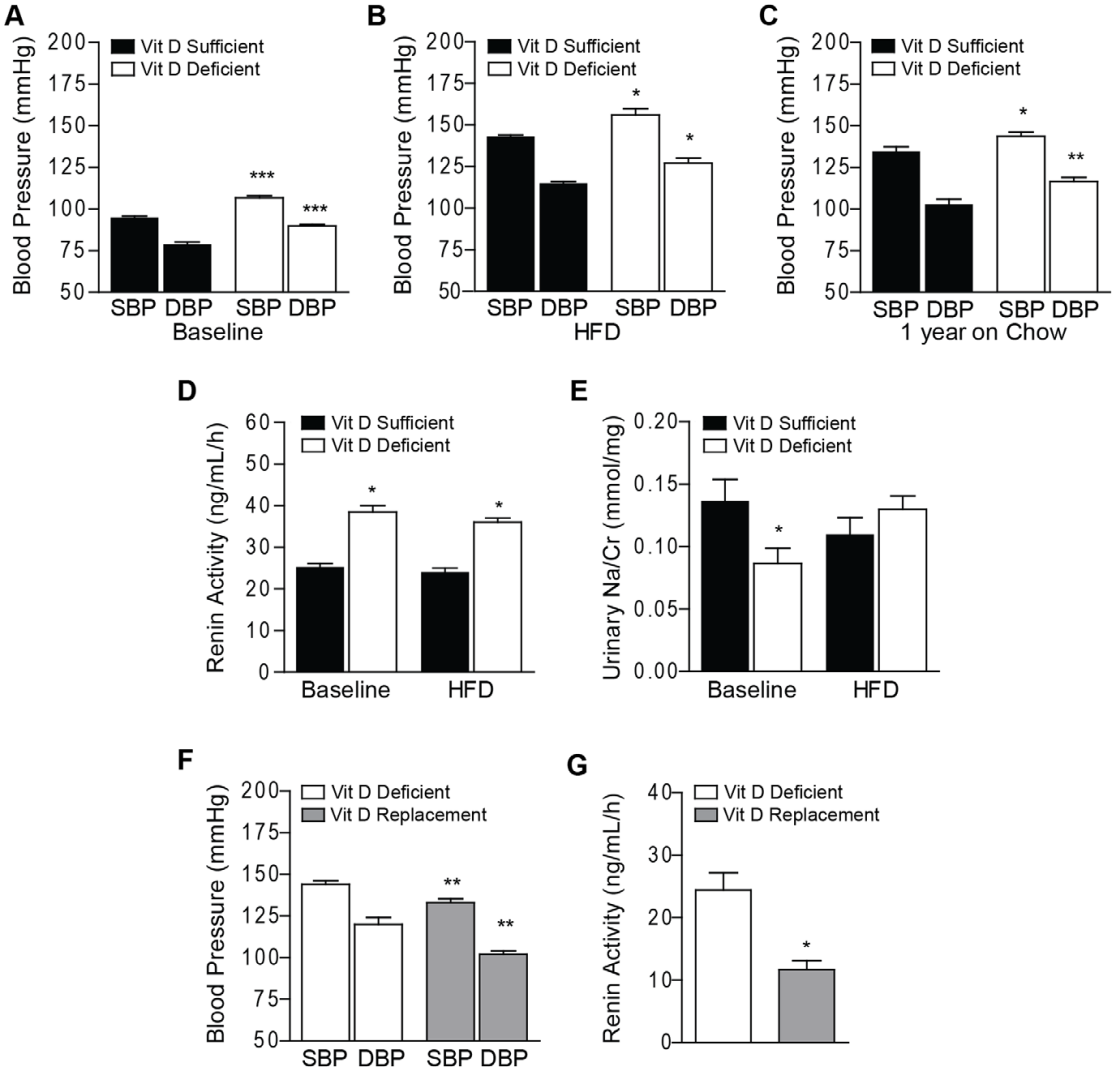
Blood pressure is reversibly increased in vitamin D deficient LDLR^−/−^ mice. Non-invasive systolic (SBP) and diastolic blood pressure (DBP) in LDLR^−/−^ mice on vitamin D-sufficient (black) or –deficient (white) diet (**A**) at baseline (n_suf_ = 12, n_def_ = 17), (**B**) after high fat diet (HFD) (n_suf_ = 13, n_def_ = 13), and (**C**) after 1 year on chow (n_suf_ = 10, n_def_ = 9). (**D**) Serum renin activity at baseline and after HFD (pooled samples of 10 animals per group). (**E**) Urinary sodium excretion at baseline and after HFD (baseline n_suf_ = 7, n_def_ = 5, HFD n_suf_ = 10, n_def_ = 13). (**F**) Blood pressure 6 weeks after returning vitamin D-deficient mice to a -sufficient diet (replacement: gray) (n_def_ = 7, n_replaced_ = 8). (**G**) Serum renin activity after returning vitamin D-deficient mice to a -sufficient diet (replacement: gray) (n_def_ = 3, n_replaced_ = 7). Data is expressed as mean ± SEM. *p<0.05, **p<0.01, ***p<0.0001.

We next evaluated non-invasive blood pressure at baseline and after HFD. Baseline LDLR^−/−^ mice on vitamin D-deficient diet had an 11 mmHg increase in SBP and DBP (p<0.0001 for both) and a 12 mmHg increase in SBP and DBP after high fat diet (p<0.04 for both) compared to vitamin D-sufficient diet (Figure 1A–B). Furthermore, BP remained elevated in vitamin D-deficient mice maintained for 1 year on vitamin D-deficient chow diet (p<0.05 for SBP, p<0.01 for DBP) (Figure 1C). Similarly, vitamin D-deficient ApoE^−/−^ mice had a 15 mmHg increase in SBP and DBP at baseline (p<0.003 for both) and a 13 mmHg increase in SBP and DBP after HFD (p<0.03 for both) (Figure S3A–B). To confirm that vitamin D deficiency induced elevated blood pressures, we performed invasive blood pressure measurements in ApoE^−/−^ mice after HFD (Figure S3C). Vitamin D deficiency increased mean arterial pressure by 14 mmHg relative to vitamin D-sufficient ApoE^−/−^ mice (p<0.04). As prior studies have implicated activation of the renin-angiotensin system as the mechanism by which the absence of vitamin D receptor signaling increases blood pressure, we next measured serum renin activity and urinary sodium excretion (Na_Ur_/Cr_Ur_). In vitamin D-deficient LDLR^−/−^ mice, serum renin activity was increased by >1.5-fold at baseline and after HFD (p<0.02 for both) when compared to vitamin D-sufficient mice (Figure 1D). Urinary sodium excretion was decreased by 37% at baseline with vitamin D deficiency but not after prolonged feeding with vitamin D-deficient high fat diet (p<0.05) (Figure 1E). These data are consistent with activation of the RAS and increased aldosterone effect on renal salt handling induced by vitamin D deficiency and aldosterone escape from sodium retention. To determine if activation of renin by vitamin D deficiency results in permanent changes in blood pressure, we returned vitamin D-deficient LDLR^−/−^ mice to standard, vitamin D-replete chow for 6 weeks (vitamin D replacement). 25(OH)D levels improved from 11.2±3.7 to 81.2±12.0 nmol/L. Following vitamin D replacement, SBP decreased by 11 mmHg and DBP by 18 mmHg (p<0.01) (Figure 1F), and renin decreased by 50% relative to mice maintained on vitamin D-deficient chow (p<0.05) (Figure 1G). These data suggest that vitamin D dynamically regulates blood pressure by modulating renin secretion.

### Vitamin D Deficiency Promotes Atherosclerosis

**Figure 2:**
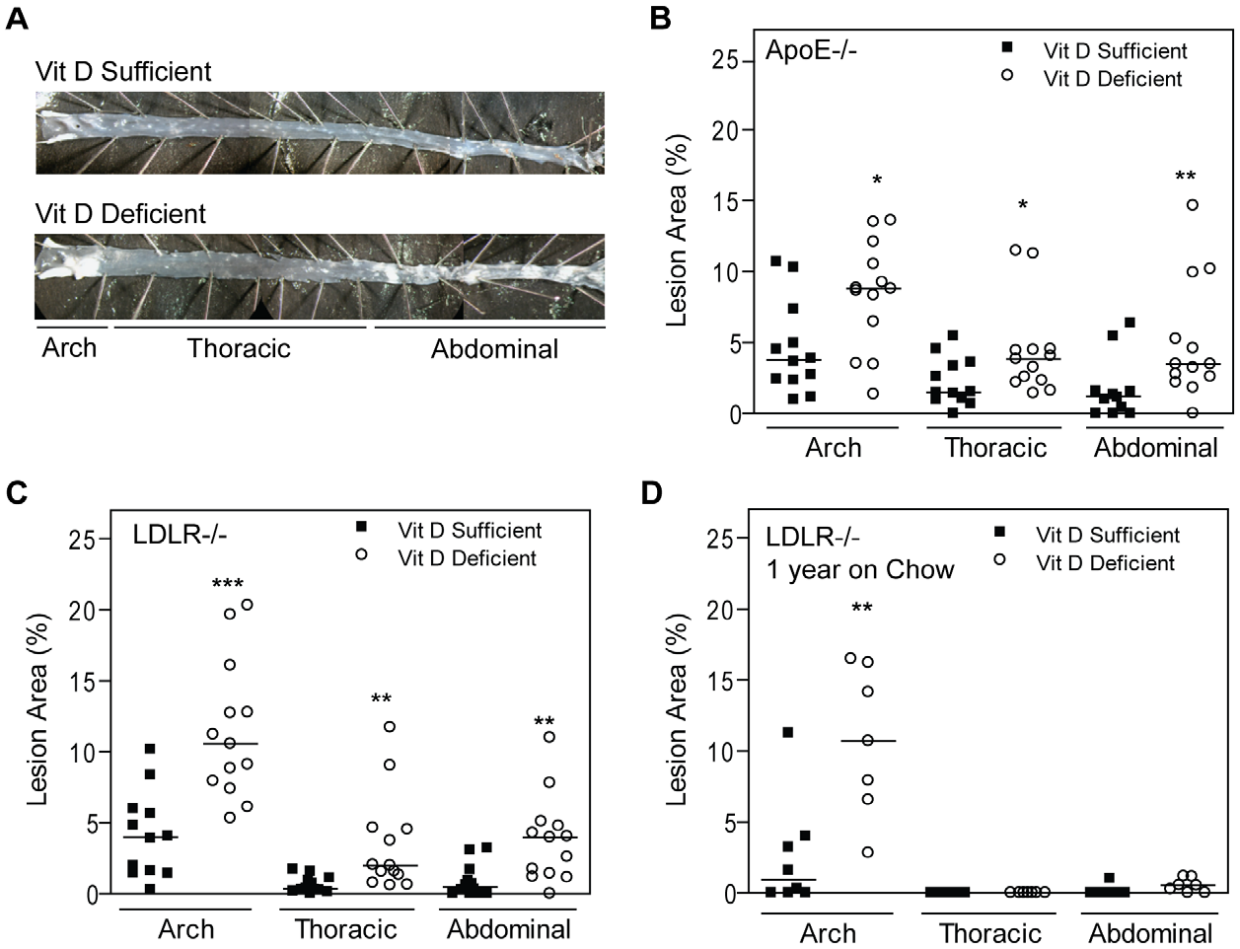
Vitamin D deficiency increases atherosclerosis. (**A**) Representative en face aortae from vitamin D-sufficient or -deficient LDLR^−/−^ mice. (**B**–**D**) Quantitative analysis of atherosclerotic lesion area in the aortic arch, thoracic and abdominal aorta for ApoE^−/−^ (n_suf_ = 12, n_def_ = 13) and LDLR−/− (n_suf_ = 12, n_def_ = 13) after HFD as well as LDLR^−/−^ mice after 1 year on chow (n_suf_ = 8, n_def_ = 7). Individual vitamin D-sufficient mice are represented as black squares and vitamin D-deficient as white circles. Median value indicated for each group. *p<0.05, **p<0.01, ***p<0.0001.

Multiple association studies in humans link vitamin D deficiency to atherosclerosis morbidity and mortality. However, it is unclear whether this nutritional deficiency is a causal factor in the development of atherosclerosis. To evaluate the effects of vitamin D deficiency, we first measured atherosclerotic lesion area in ApoE^−/−^ and LDLR^−/−^ mice fed high fat vitamin D-sufficient or -deficient diets. Vitamin D-deficient mice had increased atherosclerotic plaque area in all regions of the aorta despite identical cholesterol, triglycerides, and free fatty acids levels after HFD (Figure 2A–C). In vitamin D-deficient ApoE^−/−^ and LDLR^−/−^ mice, the median lesion area was 2.3 to 2.6-fold greater in the arch (p<0.03 for both), 2.5 to 6-fold greater in the thoracic aorta (p<0.05 for both), and 2.8 to 8-fold greater in the abdominal aorta (p<0.01 for both) when compared with vitamin D-sufficient mice in each background. Interestingly, vitamin D deficiency resulted in a 4-fold increase in plaque area in the aortic arch for LDLR^−/−^ mice maintained for one year on vitamin D-deficient compared to –sufficient chow diet without exposure to HFD (p<0.01) (Figure 2D), suggesting that this nutritional deficiency alone is critical in the acceleration of atherosclerosis particularly in aortic segments exposed to high turbulence and low shear stress.

### Vitamin D Deficiency Promotes Macrophage Lipid Accumulation

**Figure 3:**
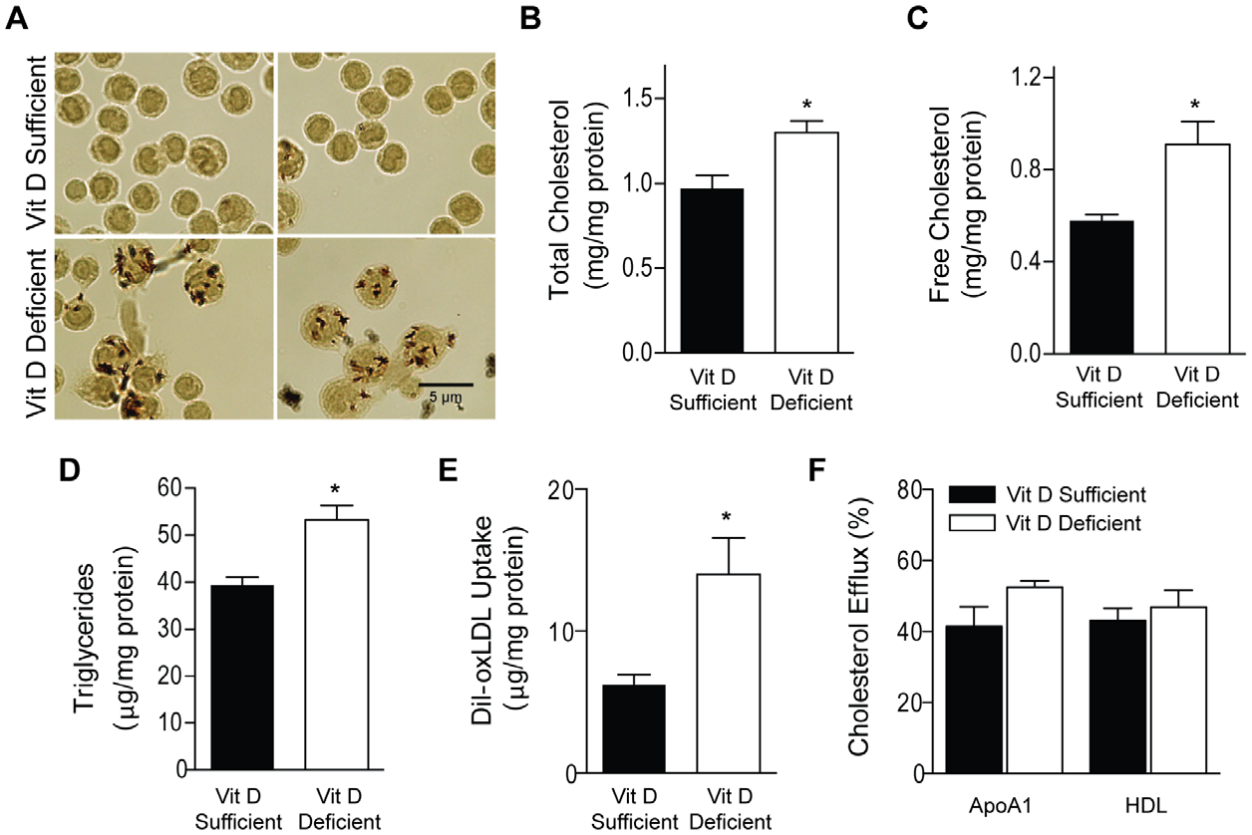
Vitamin D deficiency increases foam cell formation by altering macrophage lipid metabolism in ApoE^−/−^ mice. Peritoneal macrophages were harvested from ApoE^−/−^ mice after vitamin D –sufficient (black) or –deficient (white) HFD. (**A**) Representative Oil-Red-O stain. (**B–D**) Total cholesterol, free cholesterol, and triglyceride content (n = 3 per group). (**E–F**) Dil-oxLDL cholesterol uptake (n = 4 per group) and ApoAI-stimulated and HDL-stimulated cholesterol efflux (n = 4 per group). Data expressed as mean ± SEM. *p<0.05.

Because macrophages play an important role in vascular lipid deposition and atherosclerotic plaque development, we determined whether vitamin D deficiency affects macrophage cholesterol deposition. We obtained peritoneal macrophages from ApoE^−/−^ and LDLR^−/−^ mice after high fat vitamin D-deficient or -sufficient diet and evaluated their cholesterol content. In both ApoE^−/−^ and LDLR^−/−^ mice, vitamin D-deficient macrophages had increased foam cell formation as assessed by Oil-Red-O staining (Figure 3A, S4A) and higher total cholesterol content compared to those from -sufficient animals (p<0.04) (Figure 3B, S4B). Furthermore, vitamin D-deficient macrophages had higher free cholesterol content and triglyceride content compared to those from -sufficient mice in both mice backgrounds (p<0.05 for both) (Figure 3C–D, S4C–D). To investigate the underlying mechanism inducing macrophage cholesterol deposition by vitamin D deficiency, we assessed macrophage cholesterol uptake and efflux in peritoneal macrophages maintained in vitamin D-deficient or -sufficient conditions following extraction. Quantitation of fluorescence after fluorescence-labeled DiI-oxLDL stimulation showed that macrophages from vitamin D-deficient mice in both backgrounds had ≥30% higher oxLDL uptake compared to those from -sufficient mice (p<0.03) (Figure 3E, S4E). No differences in Apo AI- or HDL-stimulated cholesterol efflux were found between macrophages from vitamin D-deficient and -sufficient ApoE^−/−^ mice (Figure 3F–G). Thus, vitamin D deficiency accelerated foam cell formation by increasing macrophage cholesterol uptake and subsequent foam cell formation.

### Vitamin D Deficiency Alters the Macrophage Subtype in Atherosclerotic Lesions

**Figure 4:**
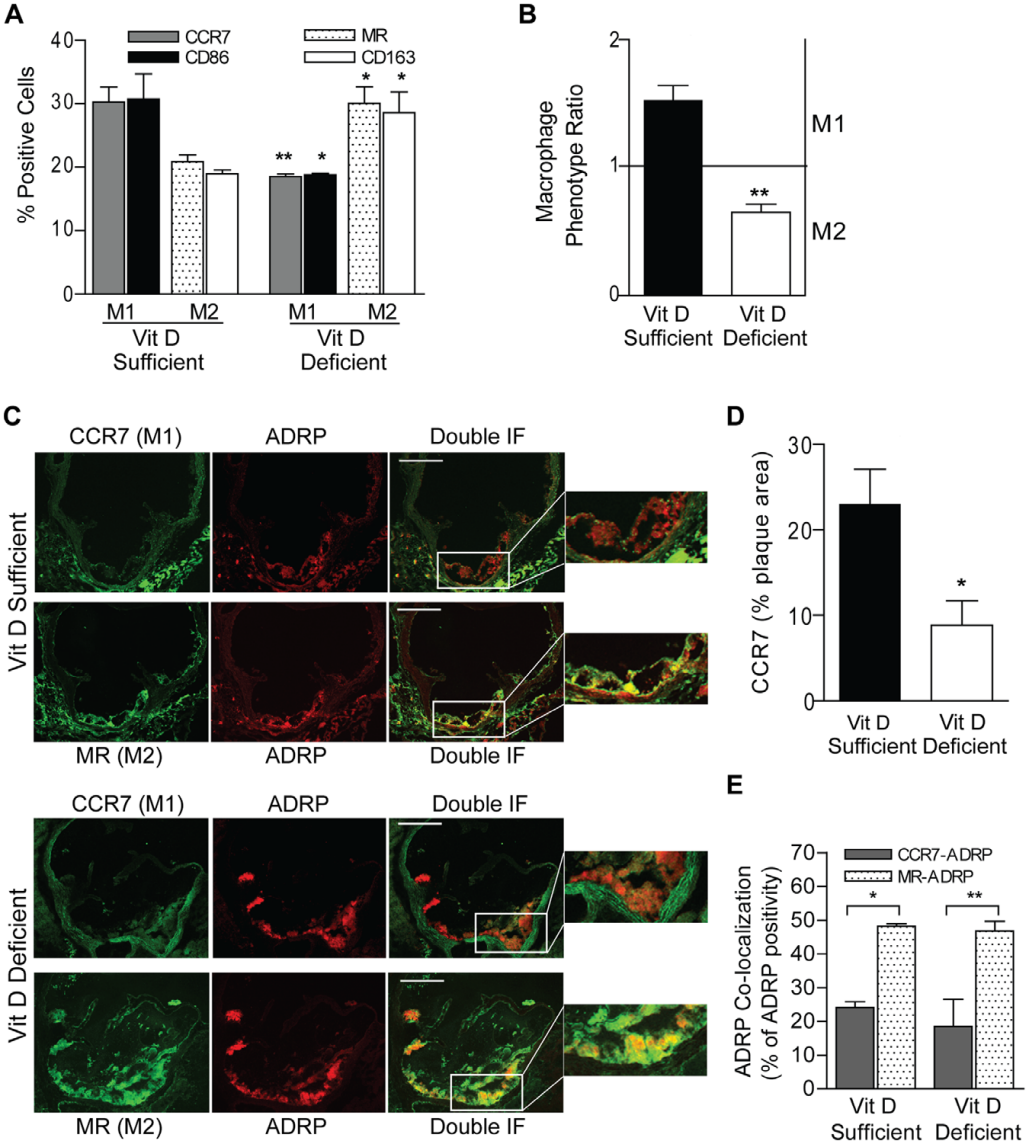
Vitamin D deficiency promotes a pro-atherogenic M2 macrophage phenotype. Peritoneal macrophages from ApoE^−/−^ mice after vitamin D-sufficient or –deficient HFD were assessed by flow cytometry for (**A**) Cell surface markers for M1 and M2 phenotype (CCR7: gray, CD86: black, MR: dots, CD163: white) and (**B**) Macrophage phenotype ratio calculated from flow cytometry analysis to assess M1 vs. M2 predominance (vitamin D-sufficient: black, vitamin D-deficient: white). From the aortic root of vitamin D-sufficient (top) and -deficient (bottom) animals after 8 weeks on HFD, (**C**) Represenative image of double immunofluorescent staining for CCR7 (M1, green), MR (M2, green), and ADRP (red). Scale bar represents 50 µm. (**D**) Quantification of CCR7 immunofluorescent staining as a percentage of total atherosclerotic plaque area, (**E**) Co-localization (yellow) of CCR7 and MR with ADRP as a percentage of ADRP-positive area (n = 3 per group for all). Data expressed as mean ± SEM. *p<0.05, **p<0.01.

Recent evidence suggests that both classically activated M1 and alternatively activated M2 macrophages are present in the atherosclerotic plaque. To begin characterizing the role of vitamin D status in macrophage differentiation in vivo, we examined the macrophage subtype of unstimulated peritoneal macrophages from ApoE^−/−^ mice by flow cytometry. Macrophages from vitamin D-deficient mice had >40% higher M2 markers (CD86 and MR) and nearly 40% lower M1 markers (CD163 and CCR7) than those from vitamin D-sufficient mice (p<0.05 for all) (Figure 4A). As a composite score of the relative balance of M1 and M2 macrophages, we calculated the Macrophage Phenotype Ratio (MPR) which we recently described for human macrophages (Figure 4B) [43]. Vitamin D deficiency resulted in an MPR<1, consistent with M2 predominance, while vitamin D sufficiency resulted in an MPR >1, consistent with M1 predominance (p<0.005). To confirm that the effects of vitamin D on macrophage phenotype also occurred within the atherosclerotic plaque, we examined frozen sections of the aortic root from ApoE^−/−^ mice on vitamin D-deficient or -sufficient HFD and assessed macrophage infiltration (Figure S5) and colocalization of M1 versus M2 macrophages with ADRP by immunofluorescence (Figure 4C). Vitamin D-deficient conditions increased macrophage vessel wall infiltration and induced a lower prevalence of M1 macrophages measured as a percentage of total plaque area compared to vitamin D-sufficient conditions, confirming the shift toward an M2–predominant macrophage phenotype in vivo with vitamin D deficiency (p<0.04) (Figure 4D). In the atherosclerotic plaque for both vitamin D conditions, M2 macrophages colocalized significantly more with ADRP than did M1 macrophages (p<0.05 for both) (Figure 4E), consistent with our observation that M2 macrophages accumulate more cholesterol. Therefore, the status of vitamin D not only alters macrophage phenotype, but also atherosclerotic plaque macrophage composition.

### Vitamin D Status Regulates Macrophage ER Stress in the Atherosclerotic Plaque

**Figure 5:**
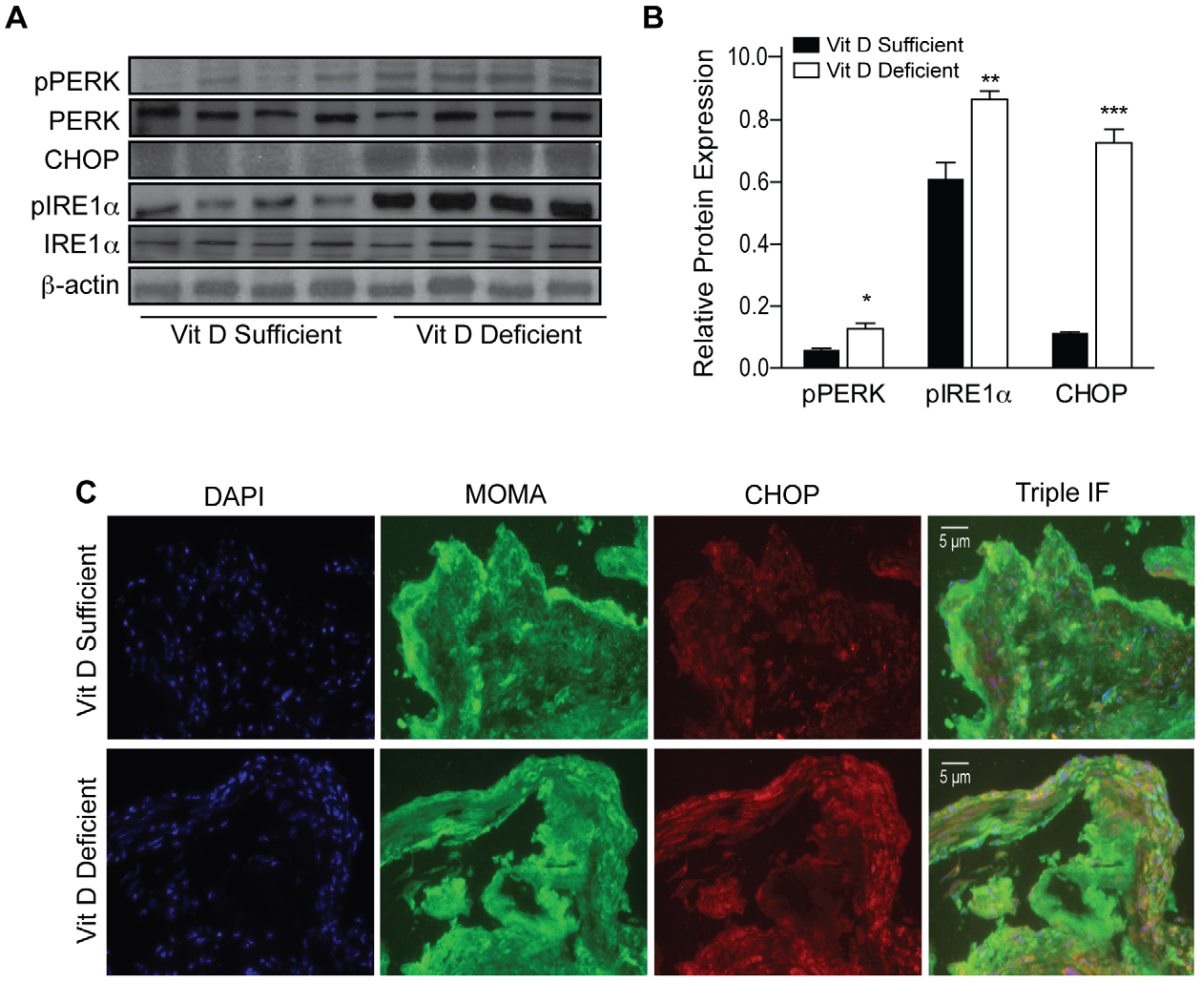
Macrophage ER stress is increased in vitamin D deficiency. ApoE^−/−^ mice were assessed after vitamin D-sufficient (black) or –deficient (white) HFD. (**A**) Western blot and (**B**) quantification of ER stress protein expression (n = 4 per group) in unstimulated peritoneal macrophages. (**C**) Triple immunofluorescent staining of the aortic root of vitamin D –sufficient (top) or –deficient (bottom) for DAPI (blue), CHOP (red), and MOMA (green). Data expressed as mean ± SEM. *p<0.05, **p<0.01, ***p≤0.0001.

Macrophage ER stress is a key regulator of both atherosclerotic plaque initiation and progression [14], [20], [21]. In patients with diabetes, active vitamin D suppresses macrophage ER stress, which shifts M2-differentiated macrophages to M1-predominant cells and decreases foam cell formation, suggesting that this hormone could be critical to atherosclerotic plaque progression in vivo. To confirm that vitamin D deficiency increases macrophage ER stress in vivo, we first assessed activated ER stress protein expression in unstimulated peritoneal macrophages from ApoE^−/−^ mice fed vitamin-deficient or -sufficient chow diets. We found that vitamin D-deficient macrophages had significantly increased activation of phospho-pancreatic ER kinase (pPERK), CHOP, and phospho-inositol requiring transmembrane kinase/endonuclease 1α (pIRE1α) when compared with macrophages from vitamin D-sufficient mice (p<0.01 for all) (Figure 5A–B). Then, we evaluated macrophage ER stress in the atherosclerotic plaque in vivo by examining frozen sections of the aortic root from ApoE^−/−^ mice on vitamin D-deficient or -sufficient HFD to determine if this increase in ER stress (CHOP in red) colocalized with macrophages (MOMA in green) within atherosclerotic plaques. CHOP staining colocalized (yellow) more with MOMA in the atherosclerotic plaque from vitamin D-deficient mice, confirming the induction of macrophage ER stress by vitamin D deficiency within the atherosclerotic plaque in vivo (Figure 5C).

### Suppression of ER Stress Prevents Vitamin D Deficiency-Induced Atherosclerosis

**Figure 6:**
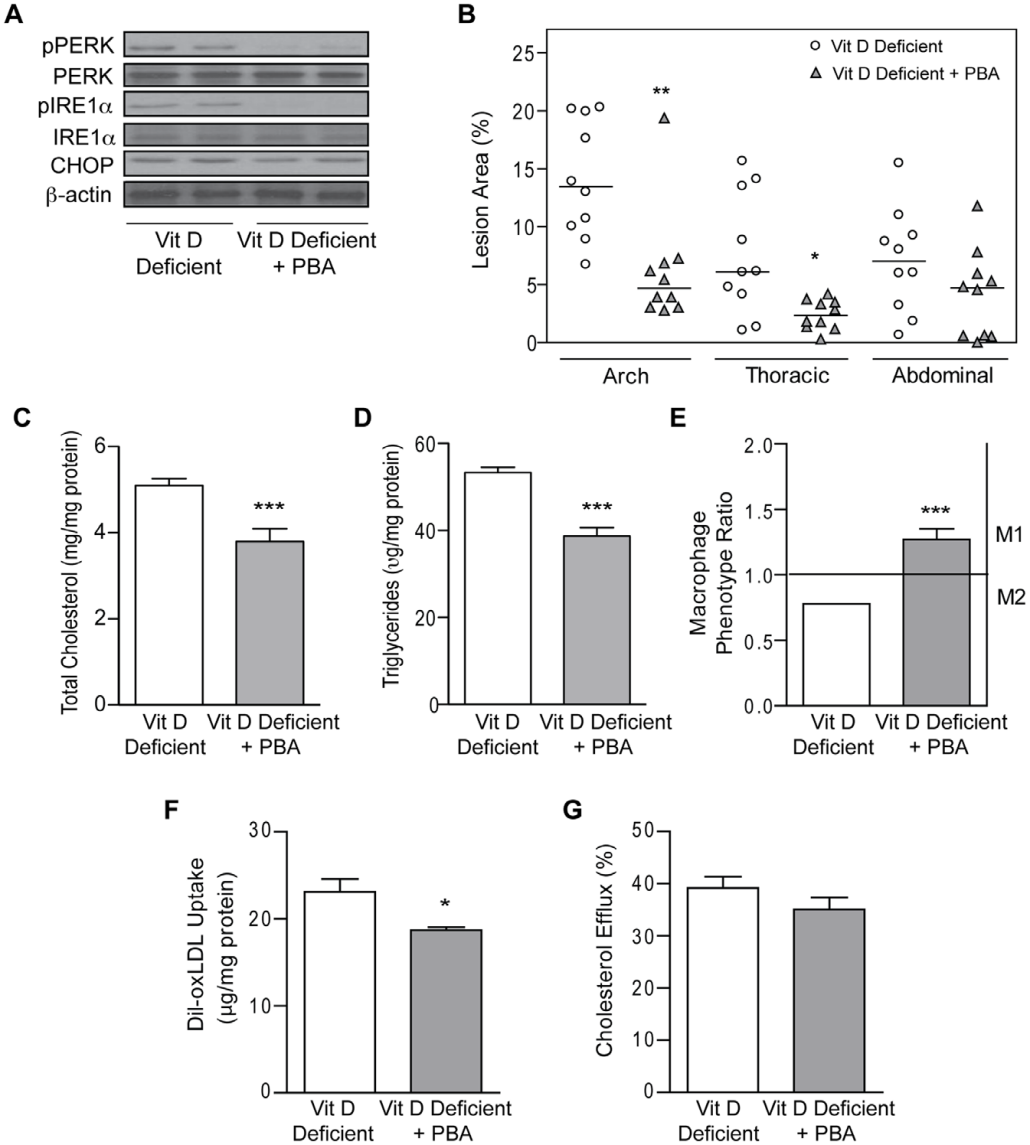
Suppression of ER stress improves cholesterol handling and reduces atherosclerosis in vitamin D-deficient mice. ApoE^−/−^ mice were assessed after vitamin D-deficient HFD with and without PBA treatment. (**A**) Western blot for ER stress protein expression in peritoneal macrophages (n = 4 per group). (**B**) Quantification of atherosclerotic lesion area for control saline-treated (white circles) and PBA-treated (gray triangles) mice (n = 10 per group). (**C–D**) Macrophage total cholesterol and triglyceride content (n = 6 per group). (**E**) Macrophage phenotype ratio based on flow cytometry analysis of cell surface markers (n = 6 per group). (**F–G**) Dil-oxLDL cholesterol uptake (n = 4 per group) and HDL-stimulated cholesterol efflux (n = 6 per group). Vitamin D-deficient data is shown in white and PBA treatment in gray. Data expressed as mean ± SEM. *p<0.05, **p<0.01, ***p≤0.0001.

To determine whether the atherosclerosis induced by vitamin D deficiency is due to activation of macrophage ER stress, we treated vitamin D-deficient ApoE^−/−^ mice with the chemical chaperone PBA, which is known to reduce ER stress, concurrently with vitamin D-deficient HFD beginning 12 weeks after weaning. After 8 weeks of PBA treatment, macrophages from PBA-treated mice showed lower ER stress protein activation compared to controls receiving only saline (p<0.05 for all) (Figure 6A, S6A) consistent with the known effect of PBA on ER stress. PBA-treated mice had no difference in plasma glucose, total cholesterol, triglycerides, free fatty acids, or blood pressure compared to non-PBA-treated vitamin D-deficient control mice (Figure S6B–F). However, PBA-treated mice demonstrated a nearly 2/3 reduction in plaque size in the arch and thoracic aorta when compared with non-PBA-treated controls (p<0.05 for both) (Figure 6B). Peritoneal macrophages from PBA treated mice had significantly lower total cholesterol and triglyceride content (p<0.0001 for both) (Figure 6C–D). In addition, suppression of ER stress with PBA shifted the macrophage phenotype toward the M1 subtype (MPR >1), while non-PBA-treated control macrophages continued to have an M2 predominance (MPR <1) (p<0.001) (Figure 6E). To determine the mechanism for the reduction of foam cell formation by PBA in vitamin D-deficient macrophages, we evaluated macrophage cholesterol uptake and efflux. Quantification of fluorescence after Dil-oxLDL stimulation showed that macrophages from PBA-treated mice had ∼20% lower oxLDL uptake compared to those from non-PBA-treated mice (p<0.03) (Figure 6F). Suppression of ER stress by PBA did not change cholesterol efflux (Figure 6G). These data suggest that vitamin D is a critical regulator of ER stress and may improve the metabolic phenotype through its effects on foam cell formation, macrophage phenotype, and atherosclerotic plaque progression.

## Discussion

Multiple studies in humans have demonstrated an association between vitamin D deficiency and hypertension and atherosclerosis. Several studies in humans indicate that replacement with vitamin D orally or by UVB reduces blood pressure. However, it has never been demonstrated that nutritional vitamin D deficiency contributes to increased blood pressure and atherosclerosis progression. Here, we demonstrate that diet-induced vitamin D deficiency increased blood pressure in mice through activation of the RAS and that vitamin D supplementation reversed this hemodynamic change by suppression of RAS activation. Furthermore, activation of macrophage ER stress by vitamin D deficiency accelerated atherosclerosis by inducing a predominance of M2 macrophages in the plaque, characterized by increased cholesterol uptake and foam cell formation. In contrast, suppression of macrophage ER stress despite vitamin D-deficient diet induced an M1-predominant macrophage profile with decreased cholesterol uptake and decreased atherosclerosis without modifying blood pressure, suggesting that multiple mechanisms are involved in the effects of vitamin D on cardiovascular disease and its risk factors.

In humans, meta-analysis of several large prospective cohorts totaling more than 1800 patients found an increased relative risk (1.76) of incident hypertension in those with 25(OH)D level <50 nmol/L (20 ng/mL) compared to those >75 nmol/L (30 ng/mL) [47], [48]. In animal models, vitamin D downregulates renin gene promoter activity independently of its effects on calcium metabolism [49]. Mice lacking the VDR exhibit hypertension and cardiac hypertrophy due to increased renin expression and plasma angiotensin II production [50]. However, it is unclear whether the less severe phenotype conferred by nutritional vitamin D deficiency affects the RAS. In this study, we confirmed in multiple mouse models that vitamin D deficiency induced hypertension by activation of the systemic RAS, facilitating sodium retention. Interestingly, after prolonged vitamin D deficiency in animals on a high fat diet, differences in sodium retention resolved despite continued RAS activation, suggesting possible aldosterone escape similar to what occurs in patients with primary hyperaldosteronism [51]. We also demonstrated that replacement of vitamin D reversed these changes, which is consistent with the results of several other interventional trials in animals and humans. In vitamin D-sufficient hypertensive rats, oral administration of vitamin D_3_ decreased blood pressure and improved endothelial cell-dependent vasodilatation [52], [53]. In an 8-week treatment study consisting of oral calcium and vitamin D_3_ replacement in elderly non-diabetic women with vitamin D deficiency, plasma 25(OH)D levels increased to ≥62 nmol/L (25 ng/mL) and SBP decreased significantly by 13 mmHg compared with the calcium-treated control group [54]. UVB exposure by skin tanning sessions increased plasma 25(OH)D levels to 100 nmol/L (40 ng/mL) and decreased blood pressure in mildly hypertensive normoglycemic patients [55]. Despite mixed results from additional interventional trials [56], our study supports the data suggesting that vitamin D replacement could be an antihypertensive therapy and, importantly, suggests that vitamin D deficiency itself is a causal factor in the development of hypertension through activation of the RAS.

Data linking vitamin D deficiency and atherosclerosis comes from large epidemiological and clinical studies [27], [28], [34], [57], [58]. A prospective study of the Framingham Offspring indicates that low vitamin D levels increase CVD risk. In middle-aged Framingham volunteers with hypertension (HTN), low 25(OH)D levels (≤15 ng/ml) increased the risk of CVD by 60% during a follow-up of 5.4 years [30]. Further evidence among individuals with T2DM in NHANES III showed that low 25(OH)D levels nearly double (OR 1.70) the likelihood of developing CVD compared with normal 25(OH)D levels [58]. In LDLR^−/−^ mice, the absence of VDR signaling (LDLR^−/−^ VDR^−/−^) accelerates atherosclerosis in the ascending aorta after 8 weeks of high fat diet, possibly by local activation of the RAS in macrophages [36]. Furthermore, LDLR^−/−^ mice fed a low vitamin D diet have more calcification of lesions in the aortic root and higher expression of osteogenic factors than mice fed a high vitamin D diet [59]. In this study using multiple murine models of diet-induced atherosclerosis, we found that vitamin D deficiency caused accelerated atherosclerosis by increasing macrophage cholesterol uptake and foam cell formation without a compensatory increase in cholesterol efflux. We previously found that in vitro, active 1,25(OH)_2_D suppresses macrophage cholesterol uptake and foam cell formation by downregulation of scavenger receptor CD36 and SRA-1 expression [35]. In contrast, the macrophages in this analysis were exposed to low 25(OH)D but normal 1,25(OH)_2_D levels in vivo and still had increased macrophage cholesterol deposition, suggesting that local production of active vitamin D from plasma 25(OH)D within the macrophage may be a key component of the effects of vitamin D on macrophage cholesterol deposition and foam cell formation and could explain the link between atherosclerosis and vitamin D deficiency in humans.

It is known that hemodynamic features play a major role in the localization of atherosclerotic lesions within the vascular tree. In areas such as the aortic arch, renal arteries, and the iliac bifurcation, the low shear stress and high turbulence from vessel curvature or branching induce hemodynamic disturbances that accelerate atherosclerotic development [60]. However, hemodynamic changes are not necessarily causative, but may simply increase susceptibility to atherosclerosis in areas of turbulent flow, while additional insults such as hypertension or hyperlipidemia induced by HFD are required for atherosclerosis to develop in areas of low turbulence. In mouse models of angiotensin II–induced hypertension or renal artery stenosis, hypertension and hyperlipidemia are synergistic to worsen atherosclerosis in both the ascending and descending aorta [61], [62]. In this study of diet-induced vitamin D deficiency, mice in both backgrounds also showed increased blood pressure and accelerated atherosclerosis in all aortic segments, but this effect was the most severe in the proximal aorta. Similarly, absence of the VDR (LDLR^−/−^ VDR^−/−^) in mice increases blood pressure and accelerates atherosclerosis [36]. Interestingly, 1 year-old LDLR^−/−^ mice fed vitamin D-deficient chow diet showed higher blood pressure, but increased atherosclerosis only in the proximal aorta and not in the thoracic or abdominal segments, similar to previous studies in chow fed LDLR^−/−^ mice [63], supporting the concept that other insults must be present, such as hyperlipidemia, to work synergistically with the effects of vitamin D deficiency (hypertension, immunomodulation, macrophage cholesterol deposition) to induce atherosclerosis in aortic areas of low turbulence.

Current literature regarding atherosclerotic plaque progression suggests that M1 macrophages are more pathogenic with a pro-inflammatory profile while M2 macrophages contribute to tissue repair [64]. However, considering the complexity of the cytokine milieu and the changes during different stages of plaque evolution, the current inflammatory paradigm may be too simplistic. Previous studies have demonstrated that macrophages can shift their differentiated phenotype back and forth from M1 to M2 under various environmental conditions during plaque evolution [65]. In chow-fed ApoE^−/−^ mice, lesion-infiltrated macrophages of young mice exhibit predominantly the M2 phenotype, while M1 macrophages are dominant in more advanced lesions of aged mice [66]. In models of plaque regression, induction of the M1 migration marker CCR7 in macrophages facilitates their egression to the lymph nodes [9], [67], while antibodies against CCR7 ligands inhibit macrophage egression, establishing a functional role for CCR7 in plaque regression [67]. In contrast, activated M2 macrophages display a reduced capacity to handle cholesterol with increased cholesterol uptake and decreased cholesterol efflux [11], [13], metabolic changes associated with inhibition of macrophage migration [68], [69]. Therefore, discovering the environmental conditions that regulate macrophage phenotype differentiation is critical to understanding atherosclerotic plaque evolution and regression [65], [66]. Recently, we found in diabetic patients that 1,25(OH)_2_D shifts M2-differentiated macrophages to M1-predominant cells with decreased foam cell formation [43] suggesting vitamin D as a key factor in atherosclerosis development; however, it is unclear if these macrophage phenotypic characteristics affect atherosclerosis progression in vivo. In this study, we demonstrate in multiple mouse models of diet-induced atherosclerosis that vitamin D status is critical to the development of atherosclerosis. Vitamin D deficiency accelerated atherosclerosis by promoting the differentiation of macrophages into the M2 subtype with high cholesterol deposition and increased cholesterol uptake. These findings suggest that vitamin D deficiency accelerates atherosclerosis by shifting plaque macrophages toward a subtype with increased cholesterol deposition and lower expression of membrane receptors that facilitate plaque egression.

Vascular ER stress is present during multiple stages of atherosclerosis development [19]. In mouse models of diet-induced atherosclerosis, multiple mechanisms of decreasing ER stress, including knockout of ER stress protein CHOP or suppression with PBA, prevent the development of atherosclerosis [24], [70]. Chronic activation of CHOP triggers macrophage apoptosis and atherosclerosis plaque instability [14]. We recently demonstrated in diabetic patients that ER stress is a key regulator of macrophage differentiation and cholesterol deposition. ER stress is required to generate the M2 phenotype through a JNK-PPARγ-dependent pathway and increases expression of scavenger receptors CD36 and SR-A1 to increase foam cell formation [25], [35]. Active vitamin D suppresses ER stress to prevent monocyte adhesion and shift M2-differentiated macrophages to M1-predominant cells with decreased foam cell formation [43], suggesting that ER stress is a critical link between cholesterol metabolism and macrophage phenotype. However, the relationship between nutritional vitamin D status and ER stress activation in vivo and the development of atherosclerosis is unknown. In this study, we found that vitamin D-deficient mice had increased atherosclerosis with plaque macrophage ER stress signaling activation and an M2-predominant phenotype when compared to vitamin D-sufficient mice. Furthermore, suppression of ER stress by PBA reduced aortic arch and thoracic atherosclerosis by decreasing macrophage cholesterol deposition, suppressing cholesterol uptake, and shifting the macrophage phenotype from an M2- to an M1-predominance despite vitamin D-deficiency. Interestingly, suppression of ER stress with PBA did not alter the hypertension induced by vitamin D deficiency or atherosclerosis in the abdominal aorta. Taken together, these data suggest that nutritional vitamin D works through a complex interplay of multiple mechanisms to improve atherosclerosis and its risk factors beyond that of ER stress activation in the vessel wall.

In summary, this work provides evidence that vitamin D deficiency is a causative factor of hypertension by stimulation of the RAS, which is reversible with vitamin D replacement. Additionally, vitamin D deficiency increased atherosclerosis by profoundly modulating the macrophage phenotype within the atherosclerotic plaque through activation of ER stress. Increased macrophage ER stress accelerated atherosclerosis by inducing a predominance of M2 macrophages, characterized by increased cholesterol uptake and foam cell formation. Interestingly, suppression of ER stress by a chemical chaperone promoted an anti-atherogenic macrophage phenotype and prevented vitamin D deficiency-induced atherosclerosis without affecting blood pressure. Thus, we suggest that vitamin D deficiency acts through multiple mechanisms, including activation of the renin angiotensin system and macrophage ER stress to contribute to the development of hypertension and accelerated atherosclerosis, highlighting vitamin D replacement as a potential therapy to reduce blood pressure and atherosclerosis. New interventional trials from our laboratory and others evaluating the influence of vitamin D replacement on atherosclerosis progression are underway.

## Supporting Information

Figure S1 **Vitamin D deficiency does not change metabolic parameters in LDLR^−/−^ mice.** Metabolic characteristics in LDLR^−/−^ mice on vitamin D-sufficient (black) or –deficient (white) diet at baseline and after 10 weeks on HFD (n_suff_ = 12, n_def_ = 17). (**A)** Animal weight and **(B)** percent body fat as assessed by MRI. Serum metabolic profiles including **(C)** glucose, **(D)** total cholesterol, **(E)** triglycerides and **(F)** free fatty acids. Metabolic characteristics in LDLR^−/−^ mice on vitamin D-sufficient (black) or –deficient (white) chow diet for 1 year (n_suff_ = 11, n_def_ = 9) including (**G)** glucose, **(H)** total cholesterol, **(I)** triglycerides and **(J)** free fatty acids. Data expressed as mean ± SEM.
(TIF)

Figure S2 **Vitamin D deficiency does not change metabolic parameters in ApoE^−/−^ mice. Vitamin D deficiency does not change metabolic parameters in ApoE^−/−^ mice.** Metabolic characteristics in ApoE^−/−^ mice on vitamin D-sufficient (black) or –deficient (white) diet at baseline and after 8 weeks on HFD (n_suf_ = 9, n_def_ = 7). (**A)** Animal weight and **(B)** percent body fat as assessed by MRI. Serum metabolic profiles including **(C)** glucose, **(D)** total cholesterol, **(E)** triglycerides and **(F)** free fatty acids. Data expressed as mean ± SEM.
(TIF)

Figure S3 **Blood pressure is increased in vitamin D-deficient ApoE^−/−^ mice.** Non-invasive systolic (SBP) and diastolic blood pressure (DBP) in ApoE^−/−^ mice on vitamin D-sufficient (black) or –deficient (white) diet at (**A**) baseline and (**B**) after HFD (n_suf_ = 14, n_def_ = 15). (**C**) Invasive blood pressures after HFD (n_suf_ = 4, n_def_ = 5). Data expressed as mean ± SEM. *p<0.05, **p<0.01.
(TIF)

Figure S4 **Vitamin D deficiency increases foam cell formation by altering macrophage lipid metabolism in LDLR^−/−^ mice.** Peritoneal macrophages were harvested from LDLR^−/−^ mice after vitamin D –sufficient (black) or –deficient (white) HFD. (**A**) Representative Oil-Red-O stain. (**B–D**) Total cholesterol, free cholesterol, and triglyceride content. **(E)** Dil-oxLDL cholesterol uptake (n = 5 per group except total cholesterol and triglycerides n_suff_ = 4). Data expressed as mean ± SEM. *p<0.05, **p<0.01.
(TIF)

Figure S5 **Vitamin D deficiency increases macrophage infiltration into the vessel wall in ApoE^−/−^ mice.** Quantification of MOMA immunofluorescent staining of the aortic root of vitamin D–sufficient or –deficient mice. Data expressed as mean ± SEM. *p<0.01.
(TIF)

Figure S6 **Suppression of ER stress does not change metabolic parameters in vitamin D-deficient ApoE^−/−^ mice.** ApoE^−/−^ mice were assessed after vitamin D-deficient HFD with (gray) and without (white) PBA treatment. (**A**) Western blot of ER stress protein expression in peritoneal macrophages. Serum metabolic profiles including **(B)** glucose, **(C)** total cholesterol, **(D)** triglycerides, and **(E)** free fatty acids (n = 16 per group). (**F**) Non-invasive systolic (SBP) and diastolic blood pressure (n_def_ = 7, n_PBA_ = 8). Data expressed as mean+SEM. *p<0.05, **p<0.01.
(TIF)
