# Supplementary figures and images for: Vitamin D Deficiency Induces High Blood Pressure and Accelerates Atherosclerosis in Mice
Source: PLoS One. 2013 Jan 22;8(1):e54625. doi: 10.1371/journal.pone.0054625 (PMC3551761; doi:10.1371/journal.pone.0054625)

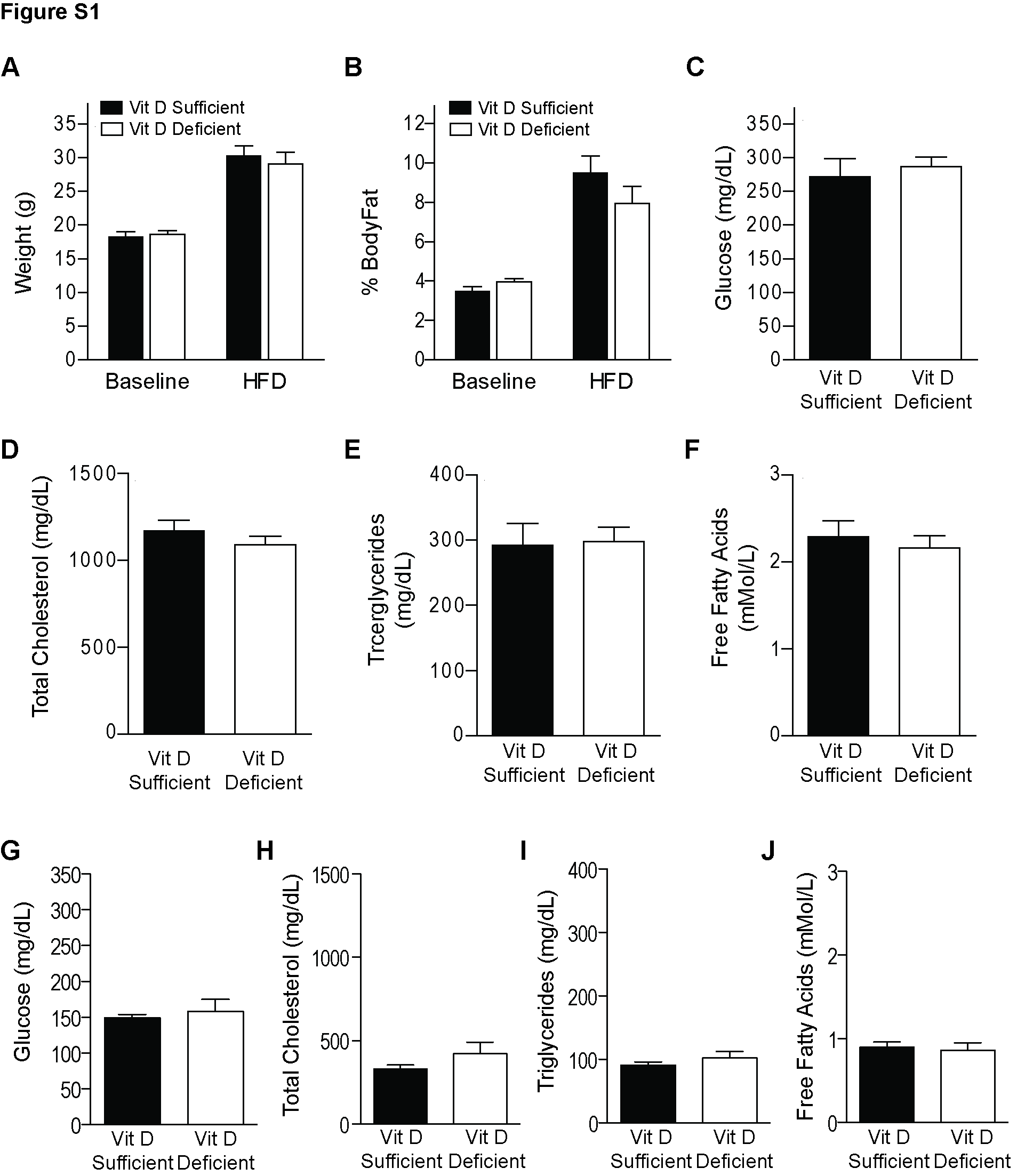

Supplement: Figure S1 — Vitamin D deficiency does not change metabolic parameters in LDLR−/− mice. Metabolic characteristics in LDLR−/− mice on vitamin D-sufficient (black) or –deficient (white) diet at baseline and after 10 weeks on HFD (nsuff = 12, ndef = 17). (A) Animal weight and (B) percent body fat as assessed by MRI. Serum metabolic profiles including (C) glucose, (D) total cholesterol, (E) triglycerides and (F) free fatty acids. Metabolic characteristics in LDLR−/− mice on vitamin D-sufficient (black) or –deficient (white) chow diet for 1 year (nsuff = 11, ndef = 9) including (G) glucose, (H) total cholesterol, (I) triglycerides and (J) free fatty acids. Data expressed as mean ± SEM. (TIF) [file pone.0054625.s001.tif]

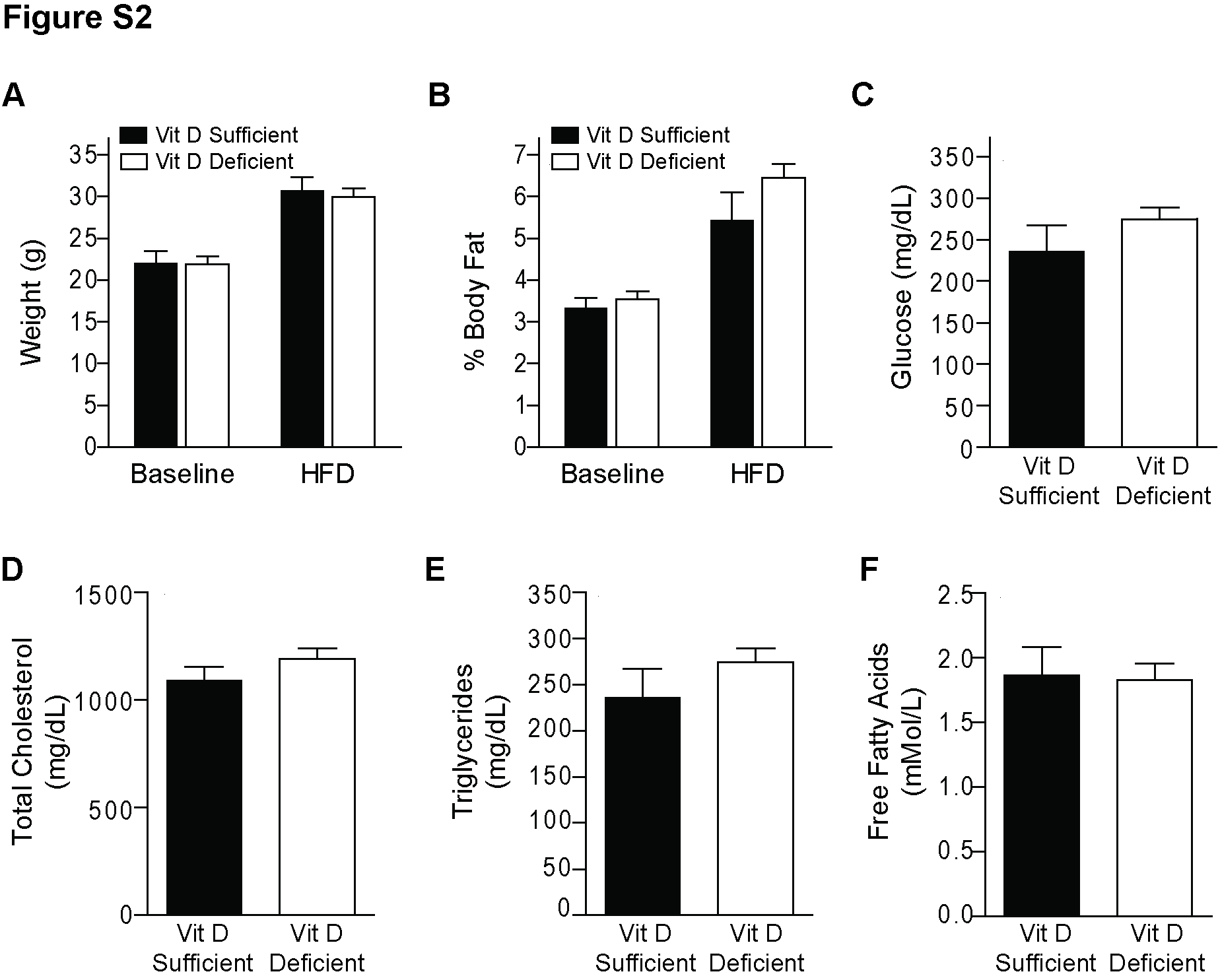

Supplement: Figure S2 — Vitamin D deficiency does not change metabolic parameters in ApoE−/− mice. Vitamin D deficiency does not change metabolic parameters in ApoE−/− mice. Metabolic characteristics in ApoE−/− mice on vitamin D-sufficient (black) or –deficient (white) diet at baseline and after 8 weeks on HFD (nsuf = 9, ndef = 7). (A) Animal weight and (B) percent body fat as assessed by MRI. Serum metabolic profiles including (C) glucose, (D) total cholesterol, (E) triglycerides and (F) free fatty acids. Data expressed as mean ± SEM. (TIF) [file pone.0054625.s002.tif]

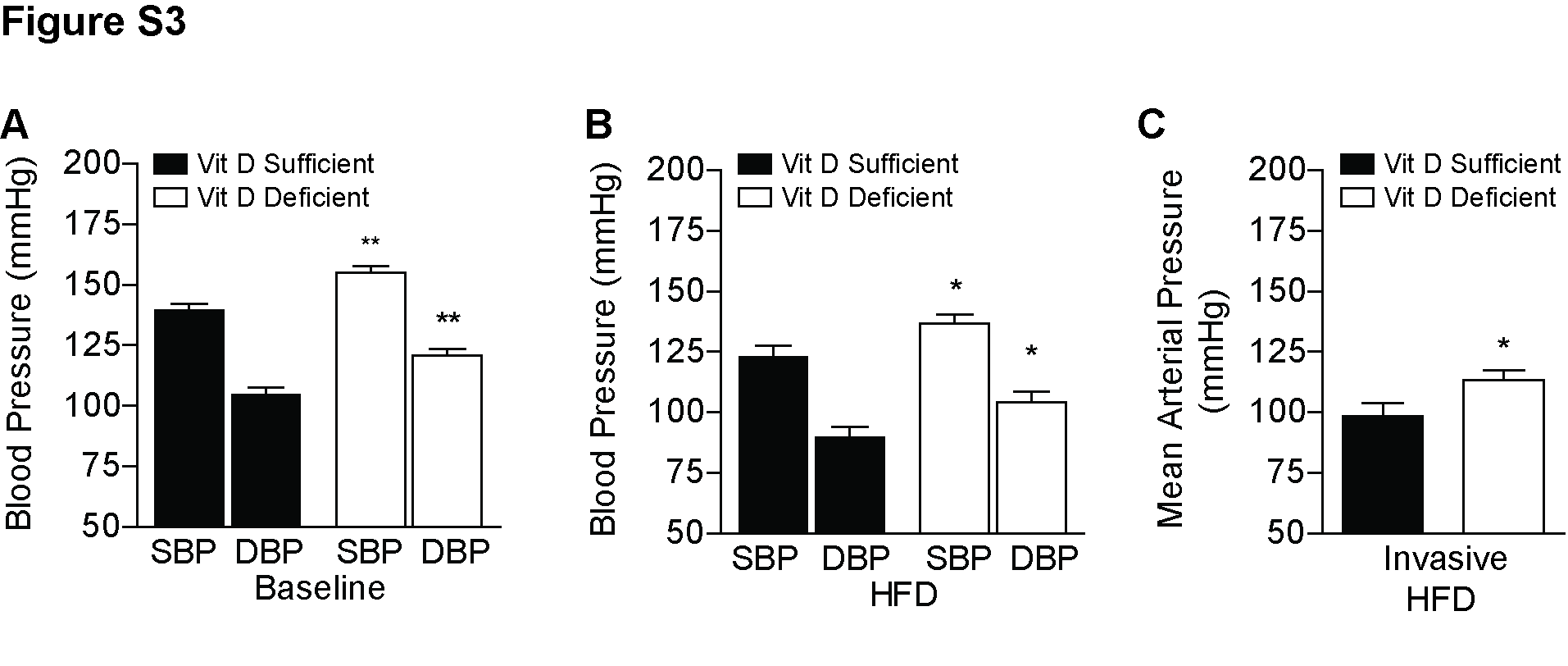

Supplement: Figure S3 — Blood pressure is increased in vitamin D-deficient ApoE−/− mice. Non-invasive systolic (SBP) and diastolic blood pressure (DBP) in ApoE−/− mice on vitamin D-sufficient (black) or –deficient (white) diet at (A) baseline and (B) after HFD (nsuf = 14, ndef = 15). (C) Invasive blood pressures after HFD (nsuf = 4, ndef = 5). Data expressed as mean ± SEM. *p<0.05, **p<0.01. (TIF) [file pone.0054625.s003.tif]

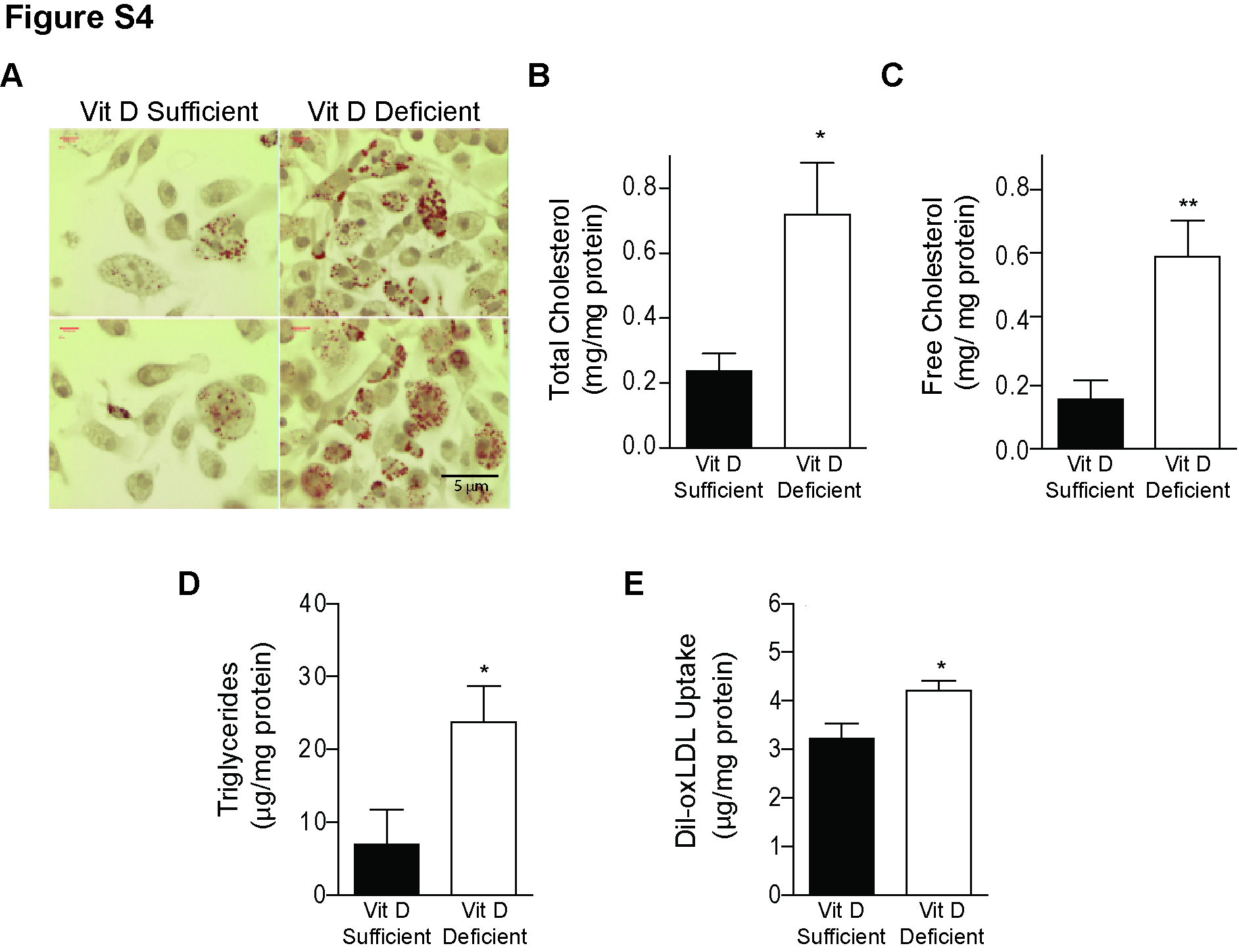

Supplement: Figure S4 — Vitamin D deficiency increases foam cell formation by altering macrophage lipid metabolism in LDLR−/− mice. Peritoneal macrophages were harvested from LDLR−/− mice after vitamin D –sufficient (black) or –deficient (white) HFD. (A) Representative Oil-Red-O stain. (B–D) Total cholesterol, free cholesterol, and triglyceride content. (E) Dil-oxLDL cholesterol uptake (n = 5 per group except total cholesterol and triglycerides nsuff = 4). Data expressed as mean ± SEM. *p<0.05, **p<0.01. (TIF) [file pone.0054625.s004.tif]

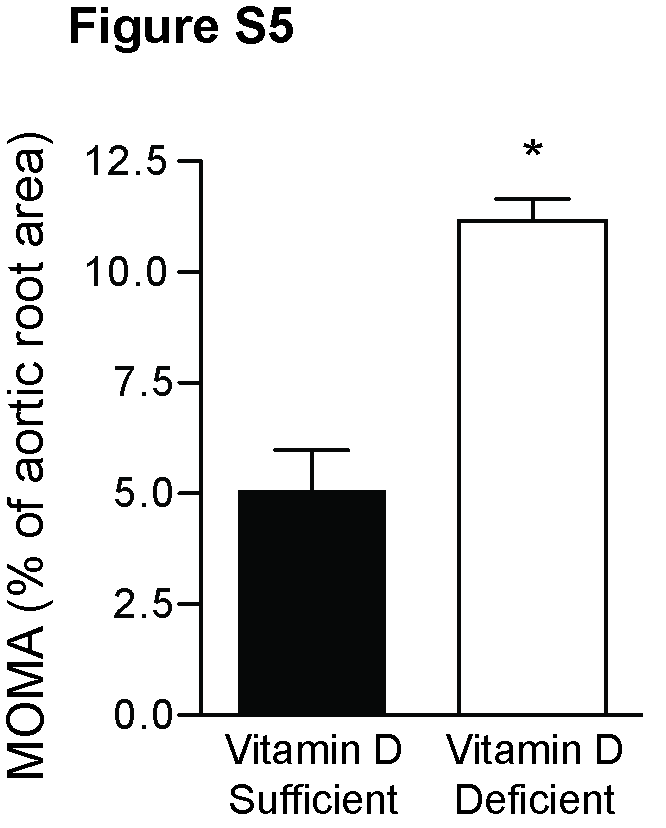

Supplement: Figure S5 — Vitamin D deficiency increases macrophage infiltration into the vessel wall in ApoE−/− mice. Quantification of MOMA immunofluorescent staining of the aortic root of vitamin D–sufficient or –deficient mice. Data expressed as mean ± SEM. *p<0.01. (TIF) [file pone.0054625.s005.tif]

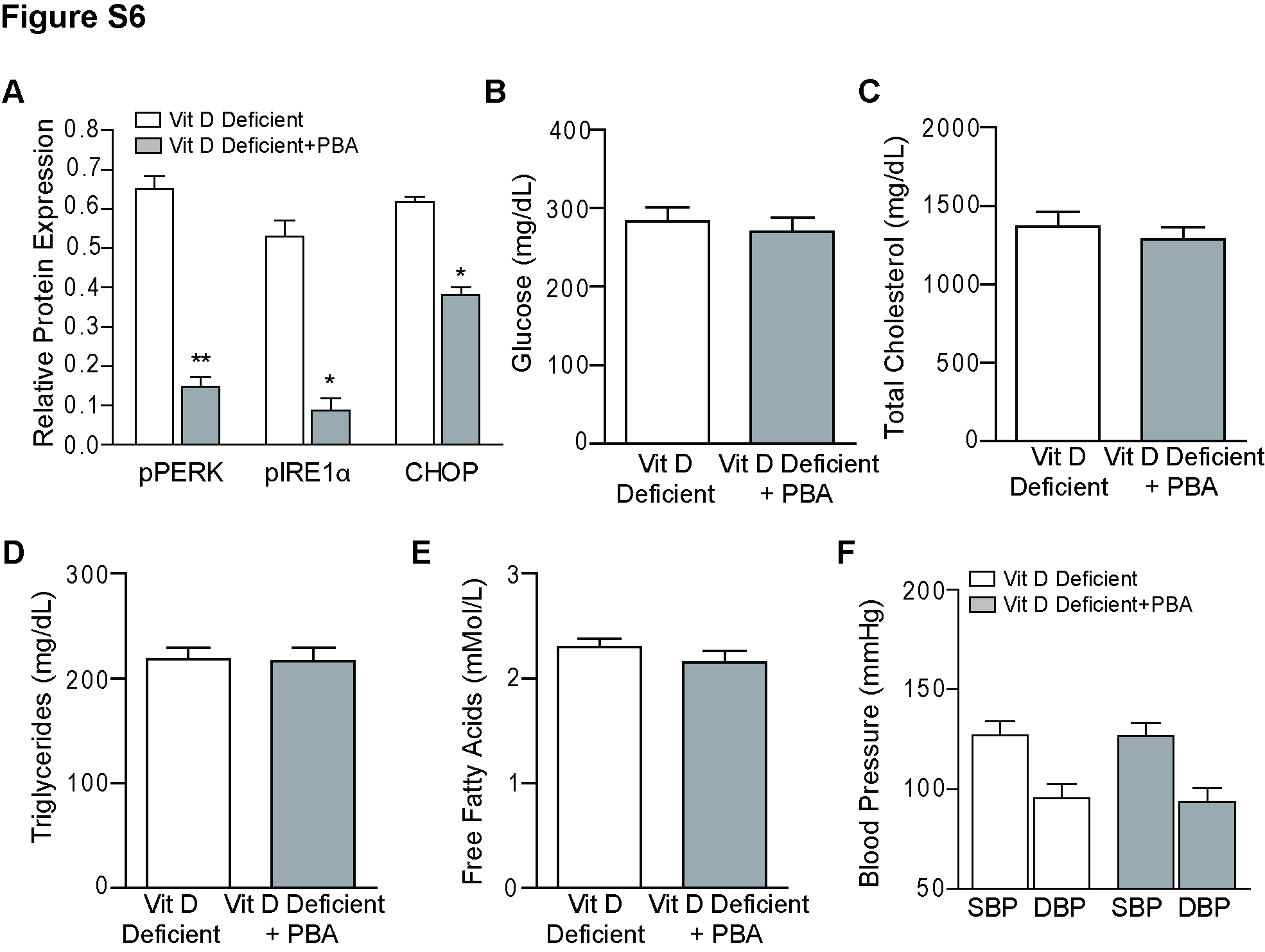

Supplement: Figure S6 — Suppression of ER stress does not change metabolic parameters in vitamin D-deficient ApoE−/− mice. ApoE−/− mice were assessed after vitamin D-deficient HFD with (gray) and without (white) PBA treatment. (A) Western blot of ER stress protein expression in peritoneal macrophages. Serum metabolic profiles including (B) glucose, (C) total cholesterol, (D) triglycerides, and (E) free fatty acids (n = 16 per group). (F) Non-invasive systolic (SBP) and diastolic blood pressure (ndef = 7, nPBA = 8). Data expressed as mean+SEM. *p<0.05, **p<0.01. (TIF) [file pone.0054625.s006.tif]
